# Wounds of Inferior Maxillary Bone

**Published:** 1890-02

**Authors:** C. E. Ballard

**Affiliations:** Saybook, Ill.


### Wounds of Inferior Maxillary Bone.

I have a case to report through the columns of the Brief. Mr. B., aged twenty-two, weight 175 pounds, plethoric, occupation sedentary. At the age of twelve, this young man received a severe blow on the inferior maxillary bone. Two or three weeks later he discovered a slight enlargement of the sublingual gland, which gradually increased in size until suppuration took place. The abscess was punctured by a physician, and upon examination of the pus, small pieces of calcareous matter was found.

The glandular opening made by the bistoury healed and the young man had no more trouble from the "still" enlarged gland until last winter, when both the sublingual and submaxillary glands enlarged and suppurated.

An opening was made in the abscess and contents removed, but the opening made by the puncture has not yet healed, and through it I have removed over fifty pieces of calcareous matter.

This young man is a perfect picture of health and his digestive powers are not impaired in the least. He has been an inveterate cornet player for ten years, and is usually in a good frame of mind.

I wish to obtain an expression in regard to the diagnosis and treatment of this case. Perhaps some of the readers of the Brief have had similar cases. I never have had one like it before. If any of the profession diagnose this condition as scrofula, I will most certainly ask for a definite explanation of what is meant by scrofula.

C. E. Ballard, M.D.

Saybook, Ill.

Incompatibility of Certain Antiseptic Substances.—The *Archives de Pharmacie* points out the incompatibility of the following: Corrosive sublimate and iodine; corrosive sublimate and soap; carbolic acid and iodine; carbolic acid and permanganate of potash; iodine and soap; salicylic acid and permanganate of potash; permanganate of potash and oil; soap and glycerine.

